# Diagnostic test accuracy of cellular analysis of bronchoalveolar lavage fluid in distinguishing pulmonary infectious and non-infectious diseases in patients with pulmonary shadow

**DOI:** 10.3389/fmed.2024.1496088

**Published:** 2024-11-20

**Authors:** Jiyang Li, Ting Wang, Faming Liu, Juan Wang, Xiaojian Qiu, Jie Zhang

**Affiliations:** ^1^Department of Respiratory, Beijing Tiantan Hospital, Capital Medical University, Beijing, China; ^2^Department of Respiratory, Chuiyangliu Hospital Affiliated to Tsinghua University, Beijing, China

**Keywords:** bronchoalveolar lavage fluid, cellular analysis, nomogram, pulmonary infectious diseases, pulmonary non-infectious disease

## Abstract

**Purpose:**

This study aims to assess the diagnostic accuracy of cellular analysis of bronchoalveolar lavage fluid (BALF) in distinguishing between pulmonary infectious and non-infectious diseases in patients with pulmonary shadows. Additionally, it will develop and validate a novel scoring system based on a nomogram for the purpose of differential diagnosis.

**Methods:**

A retrospective analysis was conducted involving data from 222 patients with pulmonary shadows, whose etiological factors were determined at our institution. The cohort was randomly allocated into a training set comprising 155 patients and a validation set of 67 patients, (ratio of 7:3), the least absolute shrinkage and selection operator (LASSO) regression model was applied to optimize feature selection for the model. Multivariable logistic regression analysis was applied to construct a predictive model. The receiver operating characteristic curve (ROC) and calibration curve were utilized to assess the prediction accuracy of the model. Decision curve analysis (DCA) and clinical impact curve (CIC) were employed to evaluate the clinical applicability of the model. Moreover, model comparison was set to evaluate the discrimination and clinical usefulness between the nomogram and the risk factors.

**Results:**

Among the relevant predictors, the percentage of neutrophils in BALF (BALF NP) exhibited the most substantial differentiation, as evidenced by the largest area under the ROC curve (AUC = 0.783, 95% CI: 0.713–0.854). A BALF NP threshold of ≥16% yielded a sensitivity of 72%, specificity of 70%, a positive likelihood ratio of 2.07, and a negative likelihood ratio of 0.38. LASSO and multivariate regression analyses indicated that BALF NP (*p* < 0.001, OR = 1.04, 95% CI: 1.02–1.06) and procalcitonin (*p* < 0.021, OR = 52.60, 95% CI: 1.83–1510.06) serve as independent predictors of pulmonary infection. The AUCs for the training and validation sets were determined to be 0.853 (95% CI: 0.806–0.918) and 0.801 (95% CI: 0.697–0.904), respectively, with calibration curves demonstrating strong concordance. The DCA and CIC analyses indicated that the nomogram model possesses commendable clinical applicability. In models comparison, ROC analyses revealed that the nomogram exhibited superior discriminatory accuracy compared to alternative models, with DCA further identifying the nomogram as offering the highest net benefits across a broad spectrum of threshold probabilities.

**Conclusion:**

BALF NP ≥16% serves as an effective discriminator between pulmonary infectious and non-infectious diseases in patients with pulmonary shadows. We have developed a nomogram model incorporating BALF NP and procalcitonin (PCT), which has proven to be a valuable tool for predicting the risk of pulmonary infections. This model holds significant potential to assist clinicians in making informed treatment decisions.

## Introduction

Pulmonary shadows are a common concern in respiratory medicine, with complex etiologies. Infectious pulmonary diseases caused by bacteria, fungi, viruses, and tuberculosis, as well as non-infectious diseases such as pulmonary edema, lung cancer, connective tissue diseases, and hematological infiltrations, can all present with pulmonary shadows (1). These conditions often share similar clinical symptoms, signs, and imaging of chest, making differential diagnosis challenging (2), with a clinical misdiagnosis rate of approximately 12% (3). Inadequate diagnosis may lead to delays in the treatment of acute conditions such as congestive heart failure and can result in unnecessary antibiotic use, contributing to antibiotic resistance (4, 5). Therefore, it is crucial to identify infectious and non-infectious pulmonary shadows early, rapidly, and accurately.

Blood leukocyte counts and classifications, C-reactive protein (CRP), and PCT are commonly used clinical indicators that can help differentiate between infectious and non-infectious pulmonary diseases to a certain extent (6, 7). However, there is currently no ideal biomarker that can accurately identify the infectious and non-infectious pulmonary diseases. Reynolds and Newball (8) introduced bronchoalveolar lavage (BAL) as a new diagnostic tool for respiratory diseases. With advancements in detection techniques, it has become evident that BALF can provide information on cytology, enzyme levels, and cytokines (9, 10). While BALF cannot serve as the gold standard for diagnosing pulmonary diseases, it can narrow the differential diagnosis and improve diagnostic accuracy. Moreover, BAL is safe, highly operable, well-tolerated by patients, and associated with a low complication rate. Consequently, BALF has become an important method for clinically diagnosing pulmonary infections. In healthy populations, the cellular composition of BALF is characterized by macrophages >85%, lymphocytes 10–15% and neutrophils ≤3%. Changes in the proportions of these cellular components hold clinical significance; for example, a neutrophil percentage exceeding 50% indicates acute lung injury, aspiration pneumonia, or purulent infections (11, 12). Although this value possesses good specificity, its sensitivity appears to be relatively low, making it inadequate for some infectious diseases with smaller involvement or milder severity. Furthermore, there is a relative lack of studies comparing the cellular analysis of BALF between infectious and non-infectious pulmonary diseases.

Consequently, the objective of this research is to perform a retrospective examination of the variations in the ratios of neutrophils and lymphocytes present in BALF from patients exhibiting pulmonary shadows attributable to both infectious and non-infectious causes. Our goal is to evaluate the diagnostic precision of cellular analysis of BALF in differentiating pulmonary infections. Furthermore, we have developed a nomogram prediction model through an extensive univariate and multivariate analysis of risk factors linked to the development of pulmonary infections. This model is designed to furnish clinicians with a swift and intuitive instrument for estimating the likelihood of pulmonary infection based on standard laboratory parameters, thereby aiding healthcare professionals in making informed treatment decisions.

## Materials and methods

### Patients

We retrospectively analyzed clinical data from patients with pulmonary shadows diagnosed in the Department of Respiratory and Critical Care Medicine at Beijing Tiantan Hospital, affiliated with Capital Medical University, between January 2022 and June 2023. The inclusion criteria were as follows: (1) confirmed to have pulmonary shadows by chest CT; (2) patients must have undergone bronchoalveolar lavage and been assessed for cellular analysis; (3) the etiology of the pulmonary shadow must be determined. The exclusion criteria included: (1) unknown etiology of the pulmonary shadow; (2) presence of two or more pathogenic infections; (3) incomplete clinical case data.

### Classification and counting method of BALF

BALF was collected following the standardized protocol recommended by the American Thoracic Society (12), with all samples processed immediately upon receipt in the laboratory. Initially, the macroscopic characteristics of the lavage fluid, including its color, transparency, and total volume, were recorded, with specific attention to the presence of mucus or debris. The lavage fluid was then filtered using sterile gauze or treated with 0.1% dithiothreitol (DTT) to dissolve mucus components. The filtered BALF was subsequently centrifuged at 4°C for 10 min, with the supernatant reserved for the analysis of soluble components. The resulting cell pellet was resuspended in 3–5 mL of 0.09% sodium chloride solution. Total cell count was determined using a hemocytometer. If the cell count exceeded the desired limit, the suspension was diluted with 0.9% sodium chloride solution to a final concentration of 5 × 10^6^ cells/mL. A 50 μL aliquot of the resuspended cell suspension was cytospin onto a glass slide. After air-drying at room temperature, hematoxylin-eosin (HE) staining was performed. The slide was first examined at low-power magnification (≤40×) under an optical microscope, and subsequently, at least 400 cells were classified and counted under high-power magnification (≥40×).

### Data collection

Clinical data from patients were gathered, encompassing (1) baseline information, including sex, age, prior cardiovascular and cerebrovascular conditions, diabetes, symptoms, and signs; (2) chest imaging conducted before and after treatment; (3) laboratory assessments: peripheral blood leukocyte counts and classifications, CRP, PCT, serological tests, antigen tests, secretion smears, cultures (sputum, BALF), pathology, metagenomic next-generation sequencing (mNGS) and additional results; (4) treatment strategy.

### Diagnosis of pulmonary shadow etiology

The determination of pulmonary shadow etiology was conducted by two infection-related professors in the department, relying on the patient’s clinical presentations, supplementary examinations including laboratory tests, chest CT, microbiological analyses, treatment strategies, and follow-up outcomes.

### Statistical analyses

Continuous variables with non-normal distributions were expressed as medians (interquartile range), and the Mann–Whitney *U* test was used to compare the pulmonary infection group with the non-pulmonary infection group. Categorical variables were presented as counts and percentages. The chi-squared (*χ*^2^) test and Fisher’s exact test were used for comparisons between groups. (LASSO) regression model was applied to optimize feature selection for the model. Then, we performed multivariable logistic regression analysis by the features selected in the LASSO regression model to identify statistically significant predictors. Based on the multivariate analysis, independent risk factors were determined, and a nomogram prediction model was established. ROC and *C*-index calibration curves were used to evaluate the model’s differentiation and prediction accuracy. The clinical utility of the model was assessed using DCA and CIC. Statistical analysis and graphics were performed using Rv.4.3.3 software.

## Results

### Comparison of baseline characteristics between two groups

**Figure 1:**
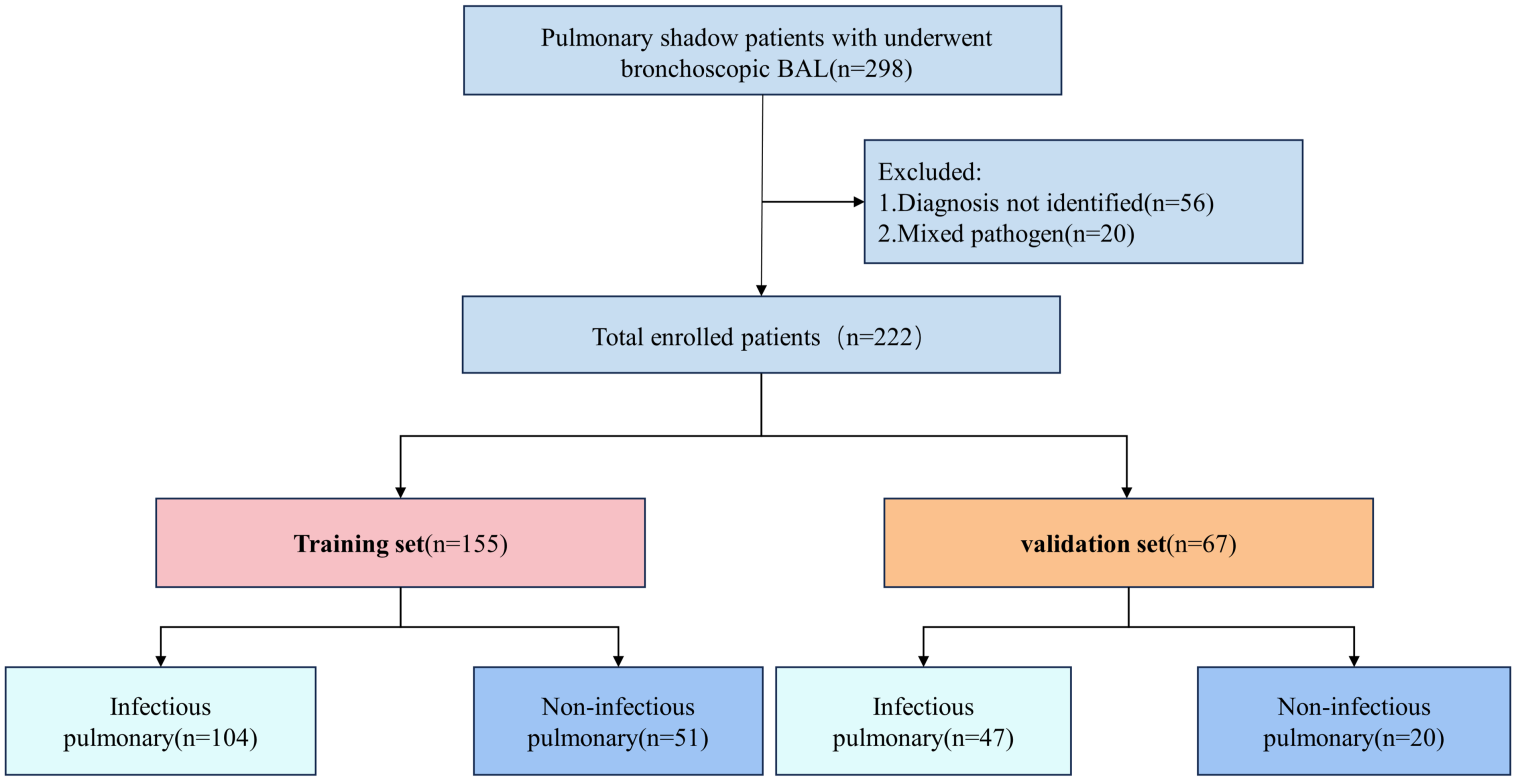
Enrollment illustration of patients with pulmonary shadow. BAL, bronchoalveolar lavage.

This study included 298 patients diagnosed with pulmonary shadows. Based on the inclusion and exclusion criteria, a total of 222 patients were selected and subsequently randomized into a training set consisting of 155 patients and a validation set comprising 67 patients, adhering to a 7:3 distribution ratio. Within the training group, 104 patients received a diagnosis of pulmonary infections, whereas 47 patients in the validation group were similarly diagnosed (Figure 1).

Analysis of baseline characteristics, including age, gender, and comorbidity, revealed no statistically significant differences between the pulmonary infection and non-pulmonary infection groups in both the training and validation cohorts (*p* > 0.05). Conversely, pulmonary infection patients exhibited significantly higher levels of leukocyte counts, neutrophil counts, CRP, PCT compared to non-pulmonary infection patients, with statistically significant differences observed (*p* < 0.05, Table 1).

**Table 1:** Baseline characteristics of the patients in the training and validation.

Variables	Training set			Validation set		
	Infectious pulmonary (*n* = 104)	Non-infectious pulmonary (*n* = 51)	*p*	Infectious pulmonary (*n* = 47)	Non-infectious pulmonary (*n* = 20)	*p*
Age, years	63.50 (50.00, 70.25)	59.00 (54.00, 69.00)	0.082	65.00 (55.50, 74.50)	62.00 (53.75, 68.00)	0.391
Male, *n* (%)	55 (52.88)	26 (50.98)	0.824	29 (61.70)	9 (45.00)	0.207
Comorbidity (%)						
Cerebrovascular disease, *n* (%)	10 (9.62)	9 (17.65)	0.152	7 (14.89)	0 (0.00)	0.165
Cardiovascular disease, *n* (%)	43 (41.35)	13 (25.49)	0.054	13 (27.66)	7 (35.00)	0.548
Chronic lung diseases, *n* (%)	19 (18.27)	7 (13.73)	0.477	9 (19.15)	3 (15.00)	0.952
Diabetes, *n* (%)	26 (25.00)	9 (17.65)	0.304	11 (23.40)	1 (5.00)	0.147
Malignant solid tumor, *n* (%)	6 (5.77)	3 (5.88)	1	1 (2.13)	2 (10.00)	0.210
Immunosuppressive state, *n* (%)	11 (10.58)	5 (9.80)	0.882	1 (2.13)	1 (5.00)	0.511
Laboratory findings						
White blood cell count (×10^9^/L)	7.89 (6.00, 12.95)	6.94 (5.75, 7.99)	**0.009**	8.51 (6.00, 11.25)	6.66 (5.55, 7.84)	**0.008**
Blood lymphocyte count (×10^9^/L)	1.40 (1.03, 1.93)	1.70 (1.32, 2.34)	**0.010**	1.40 (0.97, 1.67)	1.47 (0.97, 1.89)	0.348
Blood neutrophil count (×10^9^/L)	5.82 (3.77, 10.95)	4.03 (3.65, 4.89)	**0.001**	6.68 (4.28, 9.16)	4.37 (3.12, 5.13)	**0.002**
CRP (mg/L)	24.66 (5.93, 108.47)	3.86 (1.33, 14.97)	**0.001**	33.96 (3.77, 119.94)	6.43 (3.00, 26.06)	**0.032**
PCT (ng/mL)	0.02 (0.01, 0.07)	0.10 (0.02, 0.65)	**0.001**	0.04 (0.02, 0.08)	0.24 (0.03, 0.66)	**0.002**

CRP, C-reactive protein; PCT, procalcitonin. Values are given as the median (IQR). Statistical significance is indicated using bold typeface.

### Distribution of causes of pulmonary infectious diseases and non-infectious pulmonary diseases

A total of 151 patients were diagnosed with pulmonary infections. These included 86 cases of bacterial infections, 16 cases of fungal infections, 26 cases of branching infections, 8 cases of viral infections, and 15 cases of infections with atypical pathogens. In total, 183 strains of pathogens were identified. Among the identified pathogens, 97 were bacteria, accounting for 53% of the total. These included 57 gram-negative bacteria (31.1%) and 40 gram-positive bacteria (21.8%). Additionally, there were 29 fungi (15.8%), 20 *Mycobacterium tuberculosis* (10.9%), 16 viruses (8.7%), and 15 atypical pathogens (8.2%). Among these, 6 strains were identified as nontuberculous mycobacteria, representing 3.2% of the total (Table 2) there were 71 cases of non-pulmonary infections. These included 25 cases of lung tumors (35.2%), 15 cases of interstitial lung disease (21.1%), and 10 cases of lung sarcoidosis (14.0%).

**Table 2:** Distribution of pathogens identified in patients with infectious pulmonary disease.

Group	Pathogens	*N* (%)^a^
Bacteria (*n* = 97)	G^−^ bacteria	57 (31.1%)
	*Pseudomonas aeruginosa*	15 (8.1%)
	*Klebsiella pneumoniae*	14 (7.6%)
	*Burkholderia cepacia*	12 (6.5%)
	*Haemophilus influenzae*	7 (3.8%)
	*Mycobacterium avium*	6 (3.2%)
	*Moraxella catarrata*	3 (1.6%)
	G^+^ bacteria	40 (21.8%)
	*Streptococcus pneumoniae*	15 (8.1%)
	*Staphylococcus aureus*	9 (4.9%)
	*Enterococcus faecalis*	6 (3.2%)
	*Streptococcus agalactiae*	5 (2.7%)
	*Enterococcus faecium*	4 (2.1%)
	*Streptococcus pyogenes*	1 (0.5%)
Fungi (*n* = 29)	*Candida*	8 (4.3%)
	*Pneumocystis jirovecii*	7 (3.8%)
	*Aspergillus*	6 (3.2%)
	*Cryptococcus*	4 (2.1%)
	*Nocardia*	3 (1.6%)
	*Mucor*	1 (0.5%)
Tuberculosis (*n* = 20)	*Mycobacterium tuberculosis*	20 (10.9%)
Viruses (*n* = 16)	SARS-CoV-2	6 (3.2%)
	Influenza B virus	3 (1.6%)
	Influenza A virus	3 (1.6%)
	Cytomegalovirus	2 (1.1%)
	Herpes simplex virus	2 (1.1%)
Atypical pathogens (*n* = 15)	*Chlamydia pneumoniae*	7 (3.8%)
	*Psittacosis chlamydia*	5 (2.7%)
	*Legionella*	3 (1.6%)
NTM (*n* = 6)	*Nocardia*	4 (2.1%)
	*Actinomyces*	2 (1.1%)

a Number of patients, with the percentage in parentheses.

### Analysis of results of cellular analysis of BALF

The pulmonary infection group had a significantly higher percentage of neutrophils in the alveolar lavage fluid compared to the non-pulmonary infection group (*p* < 0.001). Conversely, the percentage of lymphocytes was significantly lower in the pulmonary infection group (*p* = 0.019, Table 3).

**Table 3:** Characteristics of BALF cellular analysis in non-infectious pulmonary disease and infectious pulmonary disease.

	Infectious pulmonary (*n* = 151)	Non-infectious pulmonary (*n* = 71)	*p*
Macrophages, (%)	17.00 (5.00, 41.50)	34.00 (23.50, 54.00)	**<0.001**
Neutrophil, (%)	50.00 (9.00, 87.00)	10.00 (3.00, 23.00)	**<0.001**
Lymphocyte, (%)	10.00 (3.00, 22.50)	20.00 (6.50, 38.50)	**0.019**

BALF, bronchoalveolar lavage fluid. Values are given as the median (IQR). Statistical significance is indicated using bold typeface.

### LASSO and multivariate analysis of risk factors associated with pulmonary infection

To assess multicollinearity among the predictors, the variance inflation factor (VIF) was calculated for each variable. The results indicated that both blood leukocyte count and blood neutrophil count had VIF values exceeding 10 (specifically 68.16 and 71.64, respectively), suggesting a significant degree of multicollinearity between these variables. Consequently, blood leukocyte count was excluded, and only blood neutrophil count was retained for further analysis. The VIF values for the percentage of neutrophils in BALF (VIF = 1.60), percentage of lymphocytes in BALF (VIF = 1.49), blood lymphocyte count (VIF = 3.45), CRP (VIF = 1.95), and PCT (VIF = 3.45) were all below 5, indicating no significant multicollinearity.

**Figure 2:**
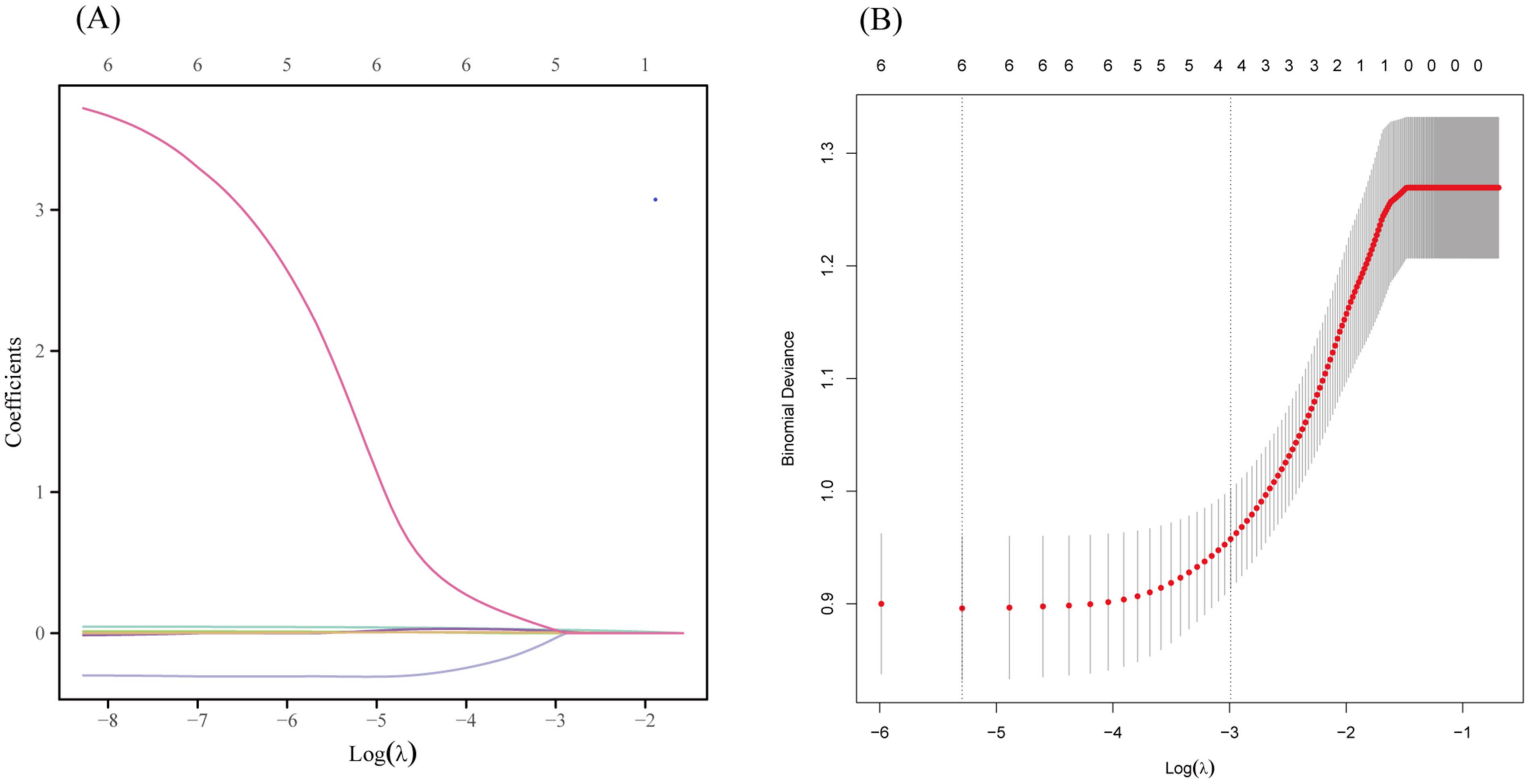
Predictors selection using the LASSO regression analysis with tenfold cross-validation. (A) Coefficient path plot for the six predictors in LASSO regression. (B) Tuning parameter (lambda) selection of deviance in the LASSO regression based on the minimum criteria (left dotted line) and the 1-SE criteria (right dotted line). In the present study predictor’s selection was according to the 1-SE criteria. SE, standard error.

Predictor selection was then performed using LASSO regression analysis with tenfold cross-validation. Initially, a coefficient path plot for the six predictors was generated (Figure 2A) to illustrate the changing trends in regression coefficients as the penalty parameter (*λ*) varied. Subsequently, the optimal *λ* value was determined based on the minimum deviance criterion (left dotted line) and the 1-SE criterion (right dotted line) (Figure 2B). In the present study, predictors were selected according to the 1-SE criterion, identifying significant factors associated with pulmonary infections, including the percentage of neutrophils in BALF, blood neutrophil count, blood lymphocyte count, and PCT. These variables exhibited non-zero regression coefficients in the LASSO model, indicating their independent contribution to predicting pulmonary infections. The selected variables were then incorporated into a multivariate regression analysis for further validation of their predictive efficacy.

**Figure 3:**
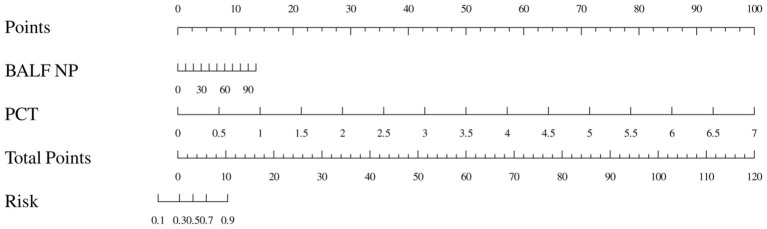
Nomogram model for predicting the occurrence of infectious pulmonary disease in pulmonary shadow patients. BALF NP, the percentage of neutrophils in BALF.

In the multivariate regression analysis, the variables identified by LASSO were included in the model. The results showed that the BALF NP (OR = 1.04, 95% CI: 1.02–1.06, *p* < 0.001) and PCT (OR = 52.60, 95% CI: 1.83–1510.06, *p* < 0.05) were independent predictors of pulmonary infections (Table 4). Using these two indicators, we established a predictive model for diagnosing pulmonary infection (Figure 3). In the Nomogram model, each factor is assigned a score displayed at the top. The total score is calculated by summing the scores of each factor. Based on this total score, a predicted risk value can be found on the last line, representing the probability that a patient will be diagnosed with lung infection.

**Table 4:** Univariate and multivariate logistic regression analysis for risk factors of infectious pulmonary disease.

Variables	Multivariate analysis	*p*
	OR (95% CI)	
BALF Neutrophils (%)	1.04 (1.02–1.06)	**<0.001**
Blood Neutrophil count (×10^9^/L)	1.00 (0.82–1.23)	0.968
Blood Lymphocyte count (×10^9^/L)	0.70 (0.34–1.44)	0.335
PCT (ng/mL)	52.60 (1.83–1510.06)	**0.021**

PCT, procalcitonin; BALF, bronchoalveolar lavage fluid. Values are given as the median (IQR). Statistical significance is indicated using bold typeface.

### Establishment and evaluation of nomogram model

**Figure 4:**
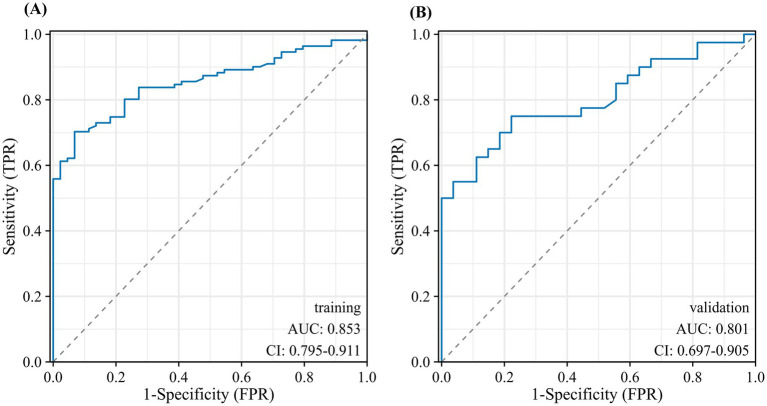
Discrimination and calibration of the nomogram model for predicting the occurrence of infectious pulmonary disease in pulmonary shadow patients. Receiver operator characteristic curve of the nomogram in the training set (A) and validation set (B).

**Figure 5:**
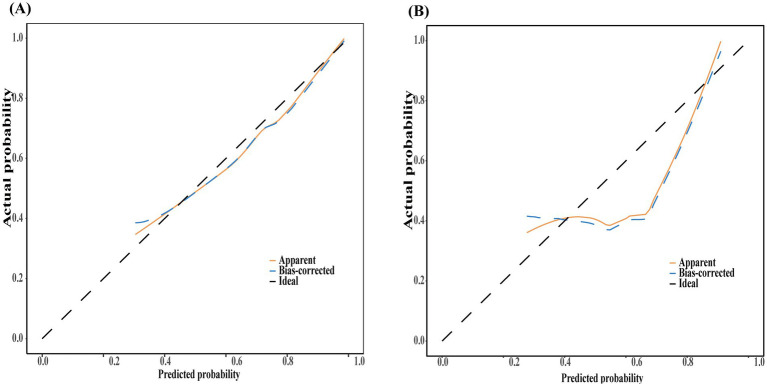
Discrimination and calibration of the nomogram model for predicting the occurrence of infectious pulmonary disease in pulmonary shadow patients. Calibration curve of the nomogram in the training set (A) and validation set (B). In these calibration curves, the *y*-axis denotes the actual observed probabilities of pulmonary infections, while the *x*-axis reflects the probabilities predicted by the nomogram. These curves serve to demonstrate the alignment between the predicted probabilities and the actual observed outcomes. The diagonal black dashed line signifies an ideal model’s perfect prediction, whereas the blue solid line indicates the predictive efficacy of the nomogram. A closer alignment of the solid line to the dashed line suggests superior predictive accuracy. AUC, area under the curve; CI, confidence interval.

**Figure 6:**
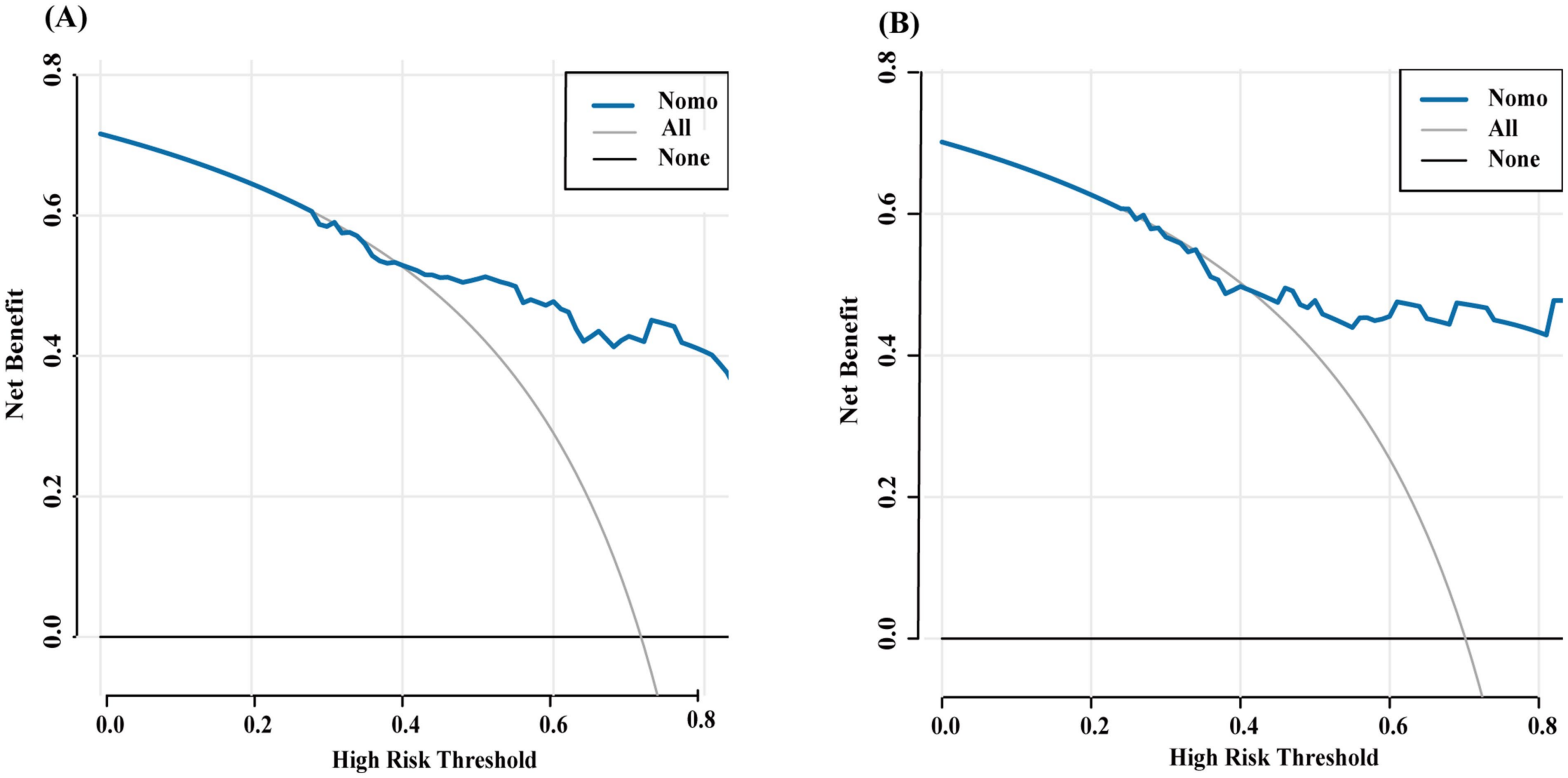
Decision curve analyses depicting the clinical net benefit of the nomogram model for predicting the occurrence of pulmonary infection in pulmonary shadow patients. The decision curve analysis is depicted for both the training set (A) and the validation set (B). The *x*-axis represents the high-risk threshold, while the *y*-axis denotes the net benefit, which is computed over a spectrum of threshold probabilities. The inclined glossy solid gray line symbolizes the scenario in which all patients are presumed to have been diagnosed with a pulmonary infection. Conversely, the horizontal solid black line reflects the scenario where none of the patients are diagnosed with a pulmonary infection. The decision curve analysis revealed that when the threshold probabilities surpassed 40%, employing the model to guide treatment decisions yielded a greater net benefit compared to treating either all patients or none, as observed in the training and validation set of this study.

**Figure 7:**
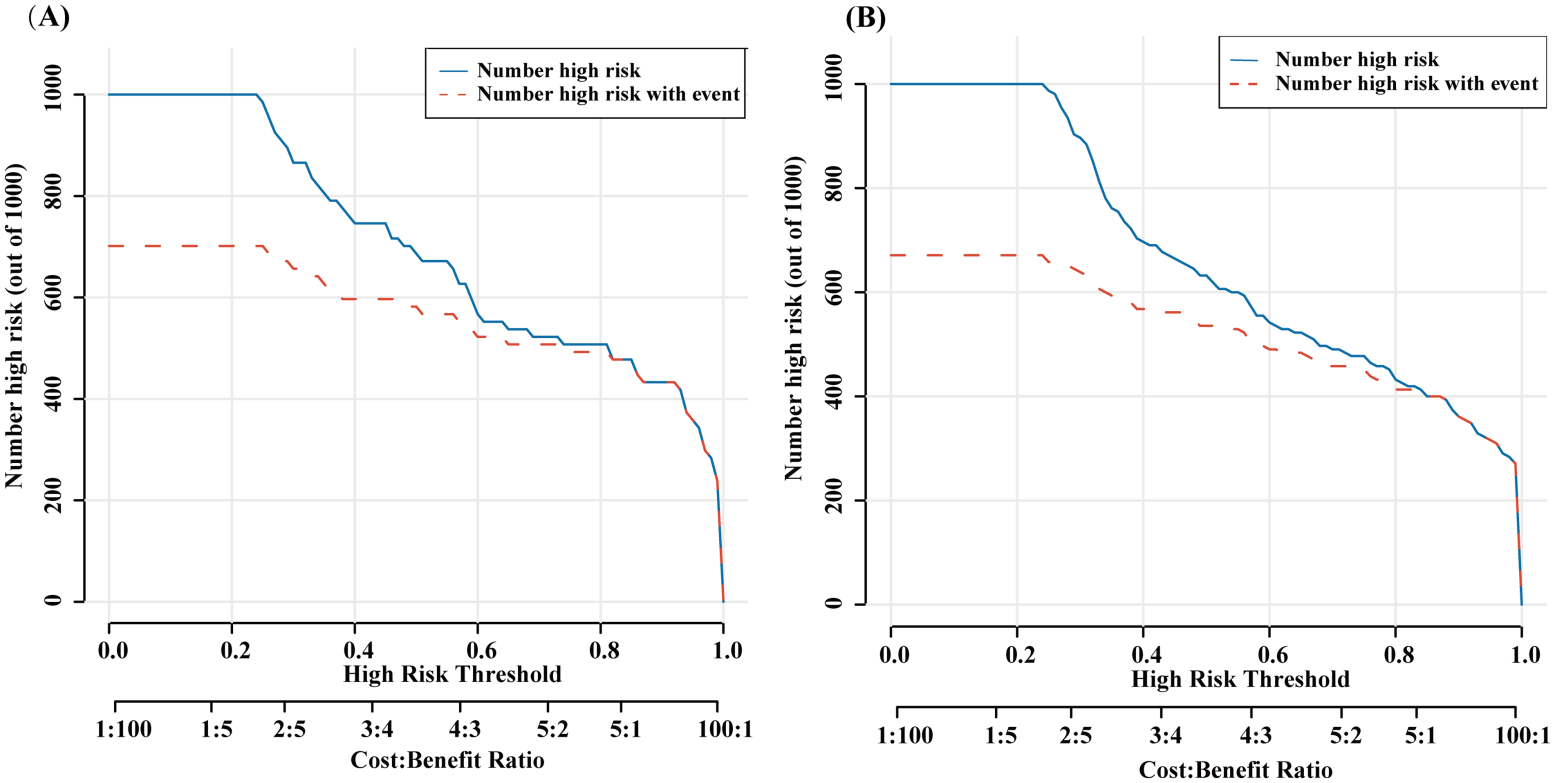
Clinical impact curve (CIC) of nomogram model in the training set **(A)** and validation set **(B)**. The red curve, representing the number of individuals classified as high-risk by the model at each threshold probability, indicates the total count of high-risk individuals. The blue curve, which denotes the number of true positives at each threshold probability, reflects the actual high-risk individuals who experienced the outcome. When the model threshold probability was ≥0.6, the model’s predictions closely align with the actual number of pulmonary infections. The CIC visually demonstrates that the nomogram provides substantial clinical net benefit and substantiates the clinical utility of the nomogram model.

The nomogram showed favorable discrimination with an AUC of 0.853 (95% CI: 0.806 to 0.918) in the training set (Figure 4A). The calibration curve demonstrates a good agreement between the model predictions and the actual observations in the training set (Figure 5A). Besides, the Hosmer–Lemeshow test yielded a nonsignificant *p* = 0.739, indicating good calibration power. The DCA results of the nomogram in the training set are presented in Figure 6A. Using this model for anti-infective treatment decisions yields greater benefits when the threshold probability is ≥40%. the CIC results of the nomogram are presented in Figure 7A. When the model threshold probability was ≥0.6, the model’s predictions closely align with the actual number of pulmonary infections.

The nomogram also demonstrated strong discrimination in the validation set, with an AUC of 0.791 (95% CI: 0.697–0.904) (Figure 4B). The calibration curve demonstrated a good agreement between the model predictions and the actual observations in the validation set (Figure 5B). In addition, the *p* = 0.376 of the Hosmer–Lemeshow indicates good calibration capability. DCA and CIC results of the Nomogram in the verification set were consistent with those of the training set (Figures 6B, 7B).

### Comparison between models

**Figure 8:**
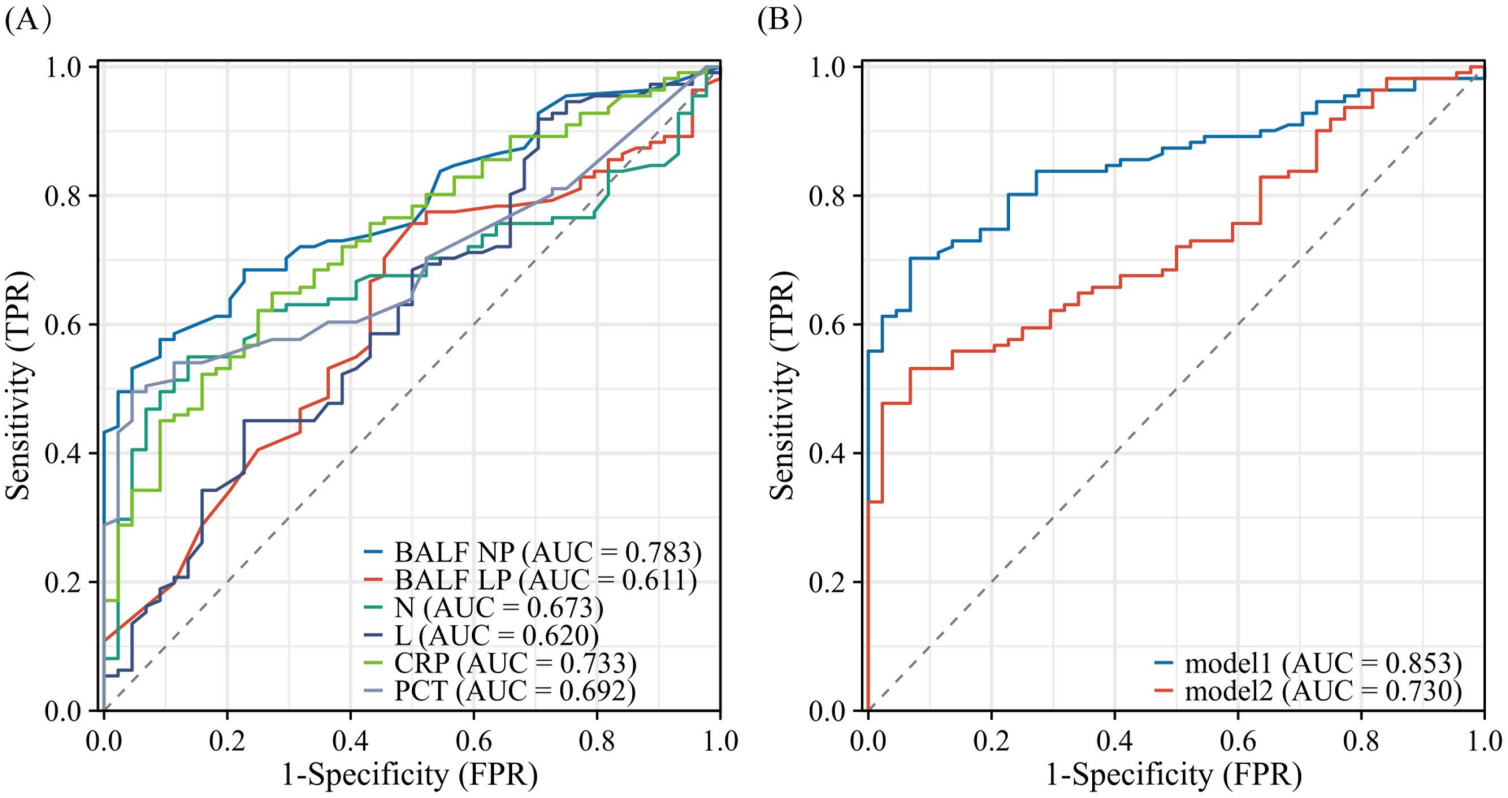
Models comparison in the whole study. Receiver operator characteristic curve of the all models (A,B) model 1: nomo, model 2: WBC + N + CRP + PCT; N: blood neutrophil count, L: blood lymphocyte count. BALF NP, the percentage of neutrophils in BALF.

BALF NP exhibited the highest area under the ROC curve (AUC = 0.783, 95% CI: 0.713–0.854), followed by CRP (AUC = 0.733, 95% CI: 0.652–0.815), PCT (AUC = 0.692, 95% CI: 0.611–0.773), the peripheral blood neutrophil count had an AUC of 0.673 (95% CI: 0.5899–0.757), the peripheral blood white blood cell count had an AUC of 0.644 (95% CI: 0.5577–0.731), and the peripheral blood lymphocyte count had an AUC of 0.620 (95% CI: 0.520–0.719). The BALF L% had an AUC of 0.783 (95% CI: 0.515–0.707). Among the individual predictive factors, BALF N% demonstrated the best discriminatory ability, achieving the highest ROC curve area (AUC = 0.783, 95% CI: 0.713–0.854). Specifically, when the BALF NP was ≥16%, the sensitivity was 72%, the specificity was 70%, the positive likelihood ratio was 2.07, and the negative likelihood ratio was 0.38 (Figures 8A,B).

**Figure 9:**
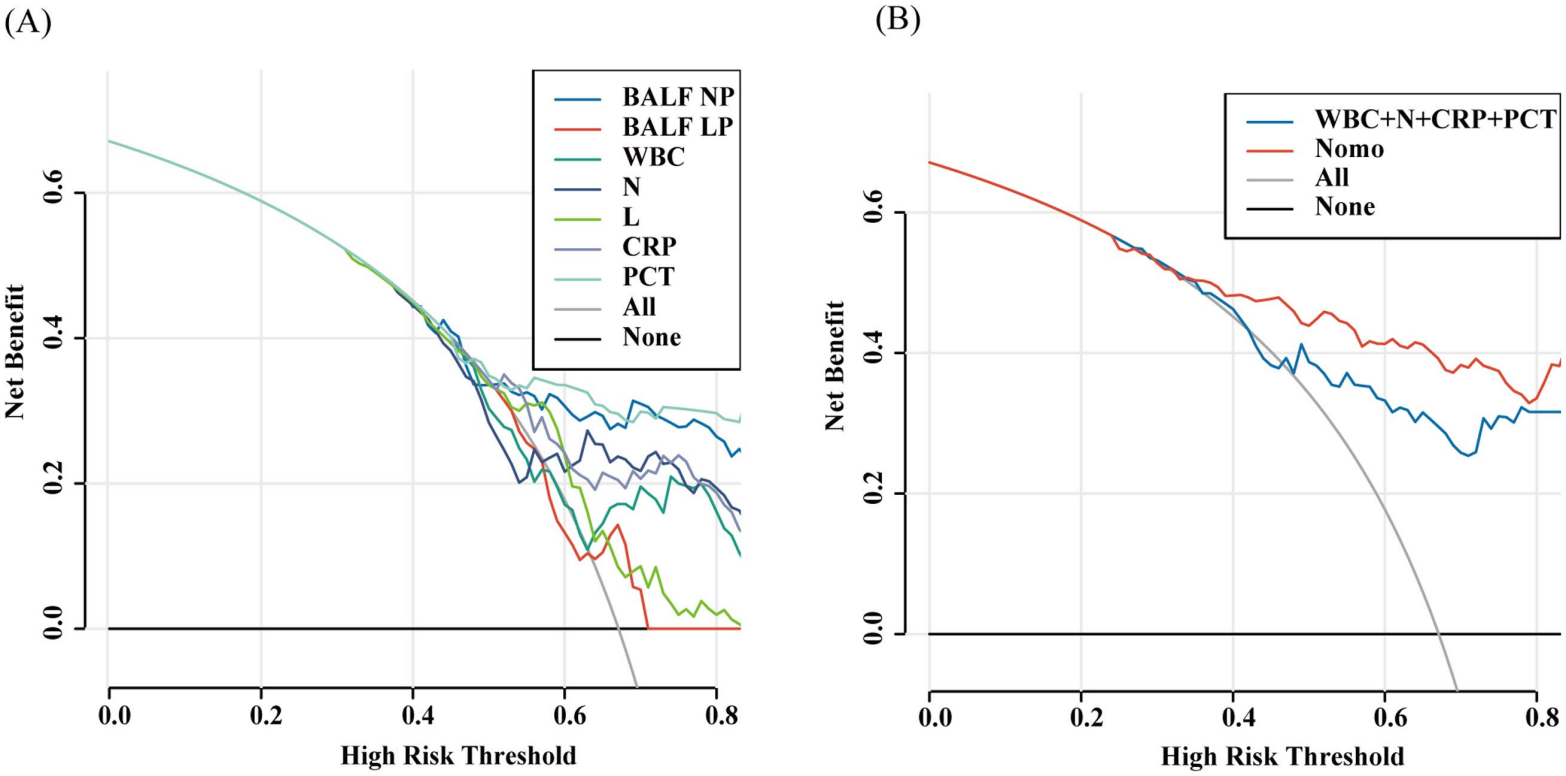
The decision curve analysis of the all models (A,B).

In the comparison of models, the Nomogram prediction model outperformed other individual predictive models, as well as the combination of peripheral blood leukocyte classification, CRP, and PCT, in predicting the risk of developing pulmonary infections across the entire range of risk thresholds (Figures 9A,B).

## Discussion

This study assessed the diagnostic accuracy of cellular analysis of BALF in distinguishing between pulmonary infectious and non-infectious diseases in patients with pulmonary shadows. The BALF NP was found to be the most accurate indicator for distinguishing between infectious pulmonary diseases and non-infectious pulmonary diseases compared to blood leukocyte counts and classifications, CRP, and PCT. Both infectious and non-infectious pulmonary diseases can lead to an elevated percentage of neutrophils in BALF. BALF NP ≥16% indicates a diagnosis of pulmonary infection with high sensitivity and specificity. The nomogram model, which uses BALF NP and PCT demonstrates good accuracy and practical applicability for diagnosing pulmonary infection.

Clinicians face challenges in identifying pulmonary infectious and non-infectious diseases in patients with pulmonary shadows. In clinical practice, the blood white blood cell count and its classification are commonly used to differentiate between infected and non-infected patients. Stolz et al. (13) analyzed the blood white blood cell count of 107 patients with immune deficiency and found that peripheral white blood cell count, peripheral blood neutrophil count and lymphocyte count could not accurately distinguish the pulmonary infectious and non-infectious diseases in patients with pulmonary shadows. Our study also supported this conclusion. CRP is also a commonly used indicator in the clinical diagnosis of pulmonary infection; however, non-infectious diseases, such as allergies and autoimmune disorders, can influence CRP levels (14, 15). In this study, we found that CRP was significantly elevated in patients with non-infectious diseases such as hypersensitivity pneumonia and connective tissue disease-associated interstitial lung disease. Consequently, the British Medical Journal highlights that the C-reactive protein assay does not have adequate sensitivity and specificity to differentiate between infiltrates on chest X-rays and the bacterial causes of lower respiratory tract infections (16). In the current study, the above indicators were poor for distinguishing between pulmonary infectious and non-infectious diseases. This may be related to the empirical use of antibiotics before admission. PCT is currently an important indicator for clinical judgment of bacterial infection, and its effectiveness in diagnosis, severity assessment, and antibiotic use for lower respiratory tract bacterial infections has been confirmed by numerous studies (17–19). However, patients with partial lung abnormalities usually present with local spots or nodules, and some severe infections may lead to multiple organ dysfunction, resulting in false negative or false positive results (6, 20). In this study, the area under the curve (AUC) value for procalcitonin (PCT) was only 0.692 (95% CI: 0.611–0.773). However, when PCT was combined with other indicators, such as the cellular analysis of BALF, the accuracy in diagnosing pulmonary infections improved significantly, resulting in an AUC value of approximately 0.853. In addition, different detection methods will also affect the results of PCT (21, 22). Therefore, at present, there is still a lack of specific and sensitive indicators to distinguish between the infectious and non-infectious pulmonary diseases.

BALF is collected from the deep bronchi, and the background biological content is significantly lower than that found in sputum. BALF is recommended by guidelines for microbiological examinations such as culture and mNGS (23). There is still limited research regarding the ability of cellular analysis to differentiate between the infectious and non-infectious pulmonary diseases. The results of this study indicate that the percentage of neutrophils serves as the best predictor for the diagnostic accuracy of pulmonary infections. An increase in neutrophil count in BALF is significantly associated with pulmonary infections, which is consistent with previous studies (24, 25). This association may be attributed to infection induced elevations of neutrophils in the alveolar interstitial space; since BALF is typically obtained from the sites of pulmonary lesions, it has a stronger capacity to respond to localized infections and inflammation (26). Additionally, antibiotic treatment has a minimal impact on neutrophil counts in BALF (27). Therefore, when the BALF NP is elevated, it is more indicative of a pulmonary infection. However, there is currently no standardized diagnostic threshold for BALF NP. Walter et al. (28) conducted an analysis of BALF cytological classification in 1,006 mechanically ventilated patients and found that a neutrophil percentage below 50% has a negative predictive value greater than 90% for bacterial pneumonia, which is significantly higher than the optimal threshold identified in this study. Pan et al. (24) assessed the role of BALF in differentiating pulmonary infection caused by different pathogens and recommended an optimal threshold of 6.7% for neutrophil percentage in BALF. However, these studies primarily focused on distinguishing between bacterial infections and non-infections, without addressing other infectious etiologies such as fungi or tuberculosis. Moreover, infections caused by different pathogens—whether bacterial, fungal, or viral—may elicit varying degrees of neutrophil elevation (25). Bacterial infections tend to show more pronounced neutrophilia, while tuberculosis infections may primarily feature either neutrophilia or lymphocytosis (29). This study mainly investigates the distinction between infections caused by different pathogens and non-infections, which is the rationale behind the proposed neutrophil cutoff value of 16% in BALF.

This study utilized both LASSO and multivariate regression analyses to identify BALF NP and PCT as independent predictors of pulmonary infection. A diagnostic model for pulmonary infection was constructed using these two indicators. ROC curve analysis revealed AUC values of 0.853 and 0.801 for the training and validation cohorts, respectively, demonstrating superior predictive performance. The calibration curve indicated a good agreement between predicted outcomes and actual observations, suggesting that the model possesses high predictive efficiency. DCA and CIC confirmed the model’s strong clinical validity. The nomogram provided a visual representation of the regression results, simplifying the interpretation of complex data compared to traditional statistical models. By illustrating the relationship between scores and outcomes, it enables clinicians to swiftly assess the risk of pulmonary infection in patients. Additionally, the diagnostic efficacy of this model outperformed the currently employed clinical markers for infection, such as peripheral blood leukocyte classification, CRP and PCT, as well as comprehensive analyses combining these three indicators.

However, there are some limitations to this study. First, it included only a laboratory features and cellular analysis of BALF. In clinical practice, clinical symptoms, signs, and imaging play crucial roles in diagnosis, and future studies should incorporate patient symptoms and imaging characteristics to enhance model accuracy. Second, as a single-center, retrospective study, the inclusion of some infectious lesions such as fungal infections and tuberculosis, as well as non-infectious conditions like acute interstitial pneumonia and specific pulmonary fibrosis, which can also elevate neutrophil counts in BALF, was limited. This may lead to potential biases in the results. Future research needs to be prospective, multicenter, and involve larger sample sizes to further validate the findings.

## Conclusion

In summary, BALF NP ≥16% effectively distinguishes between infectious pulmonary diseases and non-infectious pulmonary diseases. The predictive model based on BALF NP and PCT demonstrates excellent diagnostic performance for pulmonary infections and can assist clinicians in making informed clinical decisions.

## Data Availability

The raw data supporting the conclusions of this article will be made available by the authors, without undue reservation.
