# Corrigendum: Diagnostic test accuracy of cellular analysis of bronchoalveolar lavage fluid in distinguishing pulmonary infectious and non-infectious diseases in patients with pulmonary shadow

**DOI:** 10.3389/fmed.2025.1613520

**Published:** 2025-05-06

**Authors:** Jiyang Li, Ting Wang, Faming Liu, Juan Wang, Xiaojian Qiu, Jie Zhang

**Affiliations:** ^1^Department of Respiratory, Beijing Tiantan Hospital, Capital Medical University, Beijing, China; ^2^Department of Respiratory, Chuiyangliu Hospital Affiliated to Tsinghua University, Beijing, China

**Keywords:** bronchoalveolar lavage fluid, cellular analysis, nomogram, pulmonary infectious diseases, pulmonary non-infectious disease

In the published article, there was an error in affiliation 1. Instead of “Department of Respiratory, Beijing Tiantan Hospital Affiliated to Capital Medical University, Beijing, China,” it should be “Department of Respiratory, Beijing Tiantan Hospital, Capital Medical University, Beijing, China.”

The authors apologize for this error and state that this does not change the scientific conclusions of the article in any way. The original article has been updated.

